# Using video-taped examples of standardized patient to teach medical students taking informed consent

**Published:** 2015-04

**Authors:** SHIRIN HABIBI KHORASANI, SEDIGHEH EBRAHIMI

**Affiliations:** Department of Medical Ethics, Shiraz University of Medical Sciences, Shiraz, Iran

**Keywords:** Standardized Patient, Medical ethics, Informed consent

## Abstract

**Introduction:**

Medical student should be trained in medical ethics and one of the most essential issues in this field is taking informed consents. In this research, we compared the effect of effectiveness of teaching methods on students’ ability intaking informed consent from patients.

**Methods:**

This semi-experimental study was carried out on fifty eight subjects from the 4th-year students  of Shiraz University of Medical Sciences who attended in medical ethics coursebefore their 'clinical clerkship'training.Method of sampling was census and students were randomly allocated into two groups of control group(n=28) was trained in traditional lecture-based class and the case groupnamed as A1(n=22) were taught by video-taped examples of standardized patient.Then A1 group attended in traditional lecture-based classes named as A2. The groups were evaluated in terms the ability of recognition of ethical issuesthrough the scenario based ethical examination before and after each training. Scenarios were related to the topics ofinformed consent. Data were analyzed by SPSS 14 software using descriptive statistics and anovatest.P-Value less than 0.05 was considered as significant.

**Results:**

The mean scores results of A2, A1and B groupwere found to be7.21 , 5.91 and 5.73 out of 8,respectively. Comparison between the groups demonstrated that the ability of taking informed consent was significantly higher in A2 group (p<0.001), followed by A1group (p<0.05),while was the leastin the B group (p=0.875)

**Conclusion:**

According to this research, lecture-based teaching is still of great value in teaching medical ethics, but when combined with standardized patient, the outcome will be much better.it should be considered that mixed methodsof teaching should be used together for better result.

## Introduction

Nowadays, medical ethics is a branch of professional ethics which tries to bring ethical recommendations to the field of medical and paramedical staff (1). In the past two decades, there have been so many improvements in different fields including science and ethics. All these improvements made the traditional bioethics change to contemporary critical medical ethics. Since then, medical ethics is not just about commands and prohibitions but discusses different controversial subjects in ethical decision making (1, 2).

In developed countries, there are four principals that are used for bioethics decisions; these principals include "Autonomy", "Beneficence", "Non-maleficence" and "Justice". This theory is a very common theory in contemporary medical ethics that is used to solve problems in this field (1, 3).

Doctor-patient communication is one the important issues in professional medical ethics and includes many subjects like taking informed consent, confidentiality, giving bad news and etc. (1). On the other hand, in modern law systems, one of the most essential patient rights is the necessity of giving information while treating the patient. According to all these, in contemporary medical contracts before starting any treatment, we need patients' approval after giving them all information about success rate, risks, complications, alternative treatments and etc.. To fulfill this and with considering the principal of patients' autonomy, taking informed consent became a rule in all hospitals before any medical care, so that any invasive or non-invasive treatment (except for emergency situations) that is done without taking informed consent is equal to committing crime (1, 2).

In modern world, medical ethics has a great role in all decisions a physician should make in his/her career. Since today's world is now struggling with many ethical issues and item related to that, medical ethics has a great role in all decisions a physician should make in his/her career (4).

The physician's core ethical values are the foundation of appropriate clinical care based on a good medical relationship with the patient that effectively address patient emotions, needs, and rights. Appropriate communication between the physician and patient is a cornerstone of establishing satisfaction and providing quality health care. Therefore, teaching medical ethics and training responsible physicians and nurses is of great value. In this regard, medical students as the future physicians have certain privileges and responsibilities different from those of other students. Medical schools are responsible for ensuring that their  students are equipped with the skills required to reach the ethical standards and practice professional behavior are expected from them; therefore, training medical students is a very concerning issue. In general, medical ethics education is mostly theoretical and focuses on academic course in philosophy and ethics and unfortunately, practical clinical experiences for individuals have been very limited in medical curriculums (4). For this purpose, various methods have been developed for teaching ethics; the most common is didactic education. Among all these, a very critical field in contemporary medical ethics is taking informed consent from patients. But experience has shown that this approach alone is insufficient and will not derive efficiency of teaching. Therefore, more attention had been paid toward additional indirect methods such as using of standard patients. Over the past 30 years, using standardized patient (SP) has been significantly increased in medical education to teach and evaluate students’ communication and physical skills.

Standardized Patient (SP) is an individual who is trained to play a patient role, or is a real patient who shares his history and physical examination findings to train and evaluate medical and paramedical personnel (5). With using SP we can establish a safe and real environment so that medical/paramedical students will be able to enhance their skills without annoying real patients or making any irreparable mistakes. Also, the students can get structured feedback from their facilitators while communicating with SP(6).

From 1990, there has been a significant increase in trend to use SP in the medical curriculums. At first, there were only used for teaching clinical situations but after that, they have been also used for Objective Structured Clinical Examinations (OSCEs) to evaluate students (7). They are used in place of real patients for the OSCE since they provide a clinical scenario and help to reduce different experiences of students (6).

There are advantages and disadvantages in using SPs. One of the advantages is decreasing inter-rater variability in scoring for students. Also using SP reduces the need for physician involvement in examination. To mention disadvantages, we can say SP may not have enough skills to give appropriate feedback to students, this issue is very conflicting in literature that may be solved with doing more and reliable studies (6).

Using SP in medical ethics education is not mentioned in literatures very often and is limited to some literatures about OSCE evaluation of students and residents in some fields including psychiatry, domestic violence, taking sexual history, giving bad news, discussing “do not resuscitation “(DNR )and etc. (7).

According to this background, we conducted a comparative trial to investigate the impact of using standard patients in teaching one of the important fields in medical ethics that is taking informed consent from patients.  

## Methods

In Shiraz University of medical sciences, Duringthe 4th year of medical education, all students are required to attend a workshop of ethical issues in medicine which is almost limited to traditional lecture-based teaching in academic approach. The present semi-experimental study wastargeted fifty eight subjects from the 4th-year students which spent this course in July, 2013. This group of students were randomly divided into two treatment and control groups.Randomization achieved by employing the list of student enrolled in the course.

Informed consent was obtained from all participants prior to their participation. Considering that the participation in our study was completely voluntary some of the students did not participate in our research and those who were absent were spontaneously omitted from the study.

To use standard patient in our study we made short movies showing how to obtain informed consent from patient by a physician. The students experienced with a scheduled video-taped examples of SP. After showing the movies the students interpreted the case scenarios during a small group discussion setting led by medical ethics faculty tutors.

The Case scenarios were developed on the basis of previous work by the author that were used to elaborate different situations in medical ethics and were available in medical ethics ward. After providing these movies, they were confirmed by medical ethics experts.

Our research was an interventional study with the primary goal of evaluating the usefulness of SP encounters to medical students, to reinforce lectures on ethics and assessing whether the experience is worth adding to a lecture–based teaching. It was designed for students in the beginning of the clinical rotations. 

The group including 22 students participated in a workshop with attendance of medical ethics experts. These students are given the opportunity to observe the video-taped SP–physician interactions, portraying negative and positive examples of taking informed consents in clinical situations.

Then the participants divided into groups of 4-5 students to discuss their feelings and concerns about the patient’s views and different ethical items mentioned in the movie with each other. After that, they were examined by four scenarios which were taken out from the literature and presented to a panel of experts including white professionals and medical ethics experts with different fields of expertise and bioethics PhD students, each of them representing the requisite expertise to construct scenarios that would permit for the best extraction of ethical issues. Then they evaluated the face and content validity of the scenarios. The number of “gold standard” issues was created and agreed through a consensus of them.

The students were asked to list the issues involved in taking full informed consents in the approach to the presented patient. They also were asked to identify the strengths and shortcomings of the scenarios in the process of taking informed consents. We then assessed the responses corresponding to the fundamental ethical code and the elements of full informed consent defined in the literature of bioethics. The students were asked to respond to all scenarios and to list the ethical issues pertaining to the scenarios. 

In the next step, the 1^st^ group took part in the routine lecture-based class with the 2^nd^ group which had 28 students. The lecture-based class was finished in routine formal manner and both groups were evaluated by similar scenarios which were mentioned previously.

At last, the completed scenario questionnaires were checked and were scored according to appropriate decision making of students in the mentioned situation and considering the four key principals referred in background (Autonomy, Beneficence, etc.). Each student got a score between 0 and 8 and the scores compared for gathering proper results.

All data were analyzed by SPSS Statistics 14.0 software, descriptive statistics (mean, standard deviation),the means of score in each group were analyzed statistically by ANOVA and Tukey's test at 5 % significance level.

In our study, participation of students was completely voluntary and refusing to take part in the research had no effect in students' routine evaluation. In addition to that, all data were extracted without mentioning the students’ IDs (Name, ID number, etc.) and were all classified. 

## Results

In this study, After extracting data, 3 different groups of numbers were achieved. Group "A_1_" who were those who had taken part in SP movie workshop before participation in lecture-based class. Group "A_2_" included those students who had participated in lecture-based class after SP workshop, and the last group was those who had only taken part in lecture-based classes named Group "B".Descriptive results for each group are showed in table 1.

**Table 1:** Descriptive results for each group of medical students

Groups	Num	Min	Max	Mean±SD
Group A_1_ (SP movie)	22	2.5	8.5	5.91±1.65
Group A_2_ (SP movie+lecture)	22	5	9	7.21±1.15
Group B (lecture)	28	2	7	5.73±1.05

As it is seen in Table 1, group A2 (those who participated in both workshop and the lecture-based class) had the highest mean score; in contrast to group B (who only took part in lecture-based class) that has the least mean score. The difference between scores were calculated and the means of score in each group were analyzed statistically by ANOVA and Tukey's test at 5 % significance level. All analytical results are mentioned in the following.

The table concerning the ANOVA statistics to determine whether there is significant difference between the groups’ scores is given in table 2.

**Table 2:** One-way ANOVA test table for repetitive measures concerning the groups’ score.

Scores	Sum of squares	df	Mean square	F-statistic	Sig.
Between groups	28.625	2	14.313	8.894	0.000
Within groups	114.253	71	1.609		
Total	142.878	73			

**Table 3:** Multiple Comparisons using Tukey HSD Post Hoc Tests to see variation in mean score

Groups		Mean difference	Std. Error	Sig.	95% Confidence Interval	
					Lower Bound	Upper Bound
A1	A2	-1.25000*	0.38248	0.005	-2.1656	-0.3344
	B	.17576	0.35607	0.875	-0.6766	1.0281
A2	A1	1.25000*	0.38248	0.005	0.3344	2.1656
	B	1.42576*	0.35607	0.000	0.5734	2.2781
B	A1	-.17576	0.35607	0.875	-1.0281	0.6766
	A2	-1.42576*	0.35607	0.000	-2.2781	-0.5734

* indicates significance of mean difference at the 0.05 level.

Post hoc Tukey’s test (Table 3) showed statiscal significance between all groups. 

The greatest mean score was found in group A2, followed by groups A1 and B. Group A2 differed statistically from the other groups regarding score means [p<0.001 (group b); and p=0.005 (group A1]. No statistically significant difference was found between groups A1 and B (p=0.875).  In conclusion, comparison of two groups of A2 and B showed that in ability of taking informed consent between these two groups there is significant difference. This means that ability of taking informed consent is significantly higher in those who had taken part in both SP workshop and lecture-based class in comparison to those who only had been trained by lecture-based class. 

## Discussion

The use of standard patient appear to show an increasing trend in teaching and testing medical students from the early 1990s (7). Different studies showed that students who are trained by SP had better performance in many situations in comparison to those who hadn't the chance to educate with SP. They indicated that these students had more satisfaction of their course, more self-confidence, and more ability to interview with patients (5). In Maastricht University, all the medical students are given the opportunity to communicate with SP while learning history taking and physical examination; so that they can have better performance in real situations (5).

Using SP can be a reliable way to train medical students’ ethical performance providing teachable moments not always found in real clinical practice.

Daugherty in 1999 after reviewing literature concluded that inappropriate taking of informed consent leads to so much misunderstanding for the patients (8). Mckeague emphasized on making an environment for the patients to take proper information (9). Some other articles focused on doctor-patient relationship for taking good and proper informed consent (9, 10).

This study aimed to evaluate the impact of teaching ethics to medical students using standard patient.

As mentioned in results, the average score of group A2 –the group who had taken part in both SP movie workshop and lecture-based class- was the highest; in contrast to group B – participated only in lecture-based class – which has the least average.The averages of two groups of A1 and B were also significantly different. Several studies revealed that SP-trained students are more confident and efficient in comparison to those who are theoretically trained (5).

Our study indicated that using SP alone can play a considerable role in improving students' skill for taking informed consent. Using SP showed its better effect when it was accompanied with lecture-based class. This means that using these two methods together may enhance students' ability in learning medical ethics. Meanwhile, these two methods are being used in some universities and the results were actually more satisfying (5).A study conducted  by Sadeghi and colleagues at a psychiatric hospital showed that SP can convincingly portray psychiatric disorders and act according to the requested scenarios. According to these findings, the authors recommended the use of SP in OSCEs for psychiatric board certification exams. They also emphasized the need for the confirmation of the trained SPs by residents (11). Another study reported that OSCE is an appropriate method requiring little resources; however, it needs confirmation and feedback systems to improve its capabilities compared to the conventional examination (12). A Study by Asghari et al indicated that OSCE method alone is not enough for precise evaluation of students' ethical attitude and practice (13-15).

## Conclusion

Since education of bioethics has been almost confined to theoretical approach, we recommend usage of a practical approach for teaching informed consent and in greater value medical ethics. we designed current study to use this almost new method to improve students' skills in taking informed consent from patients. The experiments showed low score of students' ethical performance was due to lack of good training in their educational course .we could not expect them to show good ethical competency just by directlearning or by reading ethics text books alone.Therefore, indirectmethodssuch as using SP fortraining and continuous educational programs must beparts ofthe action to augment the teaching of professional ethics and interpersonal communication. Special attention should also be paidtowardsocial and multiculturaleducation.

Considering the importance of the subject, we suggest further research in this field with larger amount of participants to elaborateon the issue more.

**Limitations**

A limitation of the present study was that the intervention was made without blinding of the researcher, which has the potential for bias. Due to financial constraints in providing high technical video, the study was under-powered, and thus, did not reach statistical significance. The size, convenience, and homogeneity of the sample limit the generalizability of this study.

**Recommendations**

This study provides a number of recommendations for those who plan to investigate the advantages of Using movies as a novel teaching tool for the illustration of the medical ethical issues and integrating a case-based scenario in movies into students education context regarding medical ethics. 
